# Acid-Base Homeostasis During Vasopressin V2 Receptor Antagonist Treatment in Autosomal Dominant Polycystic Kidney Disease Patients

**DOI:** 10.1016/j.ekir.2020.12.021

**Published:** 2021-01-05

**Authors:** Judith E. Heida, Ron T. Gansevoort, Esther Meijer

**Affiliations:** 1 Department of Nephrology, University Medical Center Groningen, University of Groningen, Groningen, The Netherlands

Many patients with autosomal dominant polycystic kidney disease (ADPKD) are treated with the vasopressin V2 receptor antagonist tolvaptan to slow disease progression.^1^ By preventing binding of vasopressin to this specific receptor, tolvaptan causes a state of nephrogenic diabetes insipidus, resulting in significant polyuria, an increase in plasma sodium, and a compensatory surge in plasma vasopressin. Hypothetically, these increased levels of vasopressin set off by tolvaptan use can give rise to off-target effects via vasopressin V1 receptors, for this other type of vasopressin receptor is not blocked by this selective V2 antagonist.^2^ Potential effects of V1 activation include an increase in blood pressure via induction of vasoconstriction of the smooth muscle cells in the vascular walls, an increase of blood glucose levels by enhancing glycogenolysis in hepatocytes, an increase of glucocorticoid levels by stimulation of the adrenal cortex, and changing acid-base homeostasis in the kidneys.^3^ Considering the effect on acid-base homeostasis in particular, it should be recognized that increased V1 activation might induce a metabolic alkalosis. A recently published, meticulously executed preclinical study has demonstrated how stimulation of the V1 receptor, present on alpha-type intercalated cells in the collecting ducts of the kidneys, induces both urinary H^+^ excretion and bicarbonate reabsorption into the circulation, thus increasing plasma bicarbonate levels.^4^ In case tolvaptan indeed induces a change in the acid-base homeostasis, recognition of this state is of importance, because even a subclinical metabolic alkalosis may have consequences for a patient’s well-being. To our knowledge, the effect of tolvaptan on the acid-base balance has never been addressed in prior research.

In light of these considerations, data were collected prospectively from patients with ADPKD who started tolvaptan treatment in our hospital, the University Medical Center Groningen. Tolvaptan was initiated at a split-dose regimen of 45 mg in the morning and 15 mg in the evening, and uptitrated to 60/30 mg and finally 90/30 mg per day if tolerated. Patients had a venous blood gas drawn at baseline and at time of their maximal tolerated dose of tolvaptan. This study was designed to detect a 1.0 mEq/l change in plasma bicarbonate, which required 31 patients to be included. In addition, clinical data, such as blood pressure, and laboratory data, such as concentration of copeptin, as surrogate marker for vasopressin, were collected. The study protocol is described in detail in the supplementary methods.

These 31 patients had a mean age of 42 ± 8.3 years, 36% were men and estimated glomerular filtration rate before start of tolvaptan was 40 (33–51) ml/min per 1.73 m^2^. Mayo htTKV risk class was determined in 25 patients, of whom 5 (20%) had class 1B disease, 3 (12%) class 1C, 9 (36%) class 1D, 7 (28%) class 1E, and 1 (4%) class 2. There were no patients in class 1A. Most, namely 84% of the subjects, tolerated the daily 90/30 mg regimen, whereas 7% and 10% remained on lower dosages of 45/15 and 60/30 mg per day, respectively. As expected, V2 antagonism induced a significant polyuria with a median of 6.3 (4.41–7.20) liters per 24 hours versus 2.7 (1.75–3.34) liters on baseline (*P* < 0.001). This was accompanied by an increase in plasma osmolality from 291 (289–294) on baseline to 292 (290–297) mOsm/kg on maximal tolerated dose of tolvaptan (*P* = 0.03). Likewise, there was an increase in plasma sodium from 139 ± 1.4 mmol/l to 141 ± 2.1 mmol/l (*P* < 0.001) as well as plasma chloride from 105 ± 2.4 to 107 ± 2.2 mmol/l (*P* = 0.001). Plasma potassium did not change. Copeptin was measured as a surrogate marker for vasopressin in a small subgroup (*n* = 13). In these subjects, copeptin increased from 7.9 (4.9–25.4) to 41.0 (21.2–42.8) pmol/l (*P* = 0.001). Estimated glomerular filtration rate decreased significantly, as described previously.^5^ Evaluating the results of the venous blood gas measurement, we observed no change in bicarbonate levels (26.9 mmol/l on baseline and 27.2 mmol/l on maximal tolerated dose of tolvaptan, *P* = 0.45), but we did find a small, but statistically significant change in blood pH from 7.36 to 7.34 (*P* = 0.01), in concurrence with a significant change in pCO_2_ from 6.41 kPa to 6.71 kPa (*P* = 0.04). Both anion gap and base excess remained unchanged. In addition, blood pressure and HbA1c on baseline and at maximal tolerated dose were compared, as secondary markers for the strength of the V1 receptor-mediated changes. Including only those patients on a stable antihypertensives regimen (*n* = 19), we found no change in systolic blood pressure (128.3 ± 11.2 vs. 127.6 ± 12.7 mm Hg, respectively, *P* = 0.74) or in diastolic blood pressure (80.8 ± 8.3 vs. 80.4 ± 12.4 mm Hg, respectively, *P* = 0.83). HbA1c did also not change in those patients for whom this measure was available (*n* = 8, 35 [32.5–38.5] and 35 [30.5–38], respectively, *P* = 0.71). All data are summarized in Table 1.

**Table 1:** Effect of V2 antagonism on acid-base homeostasis: baseline versus maximal tolerated dose of tolvaptan (*n* = 31)

Physical examination	Baseline	Maximal tolerated dose	*P* value
Systolic blood pressure, mm Hg	128.3 ± 11.2	127.6 ± 12.7	0.74
Diastolic blood pressure, mm Hg	80.8 ± 8.3	80.4 ± 12.4	0.83
**Biochemical analyses**			
Osmolality, mOsm/kg	291 (289–294)	292 (290–297)	0.03
eGFR, mL/min per 1.73 m^2^	40 (33–51)	37 (30–47)	0.006
HbA1c, mmol/mol	35 (32.5–38.5)	35 (30.5–38)	0.71
Sodium, mmol/l	139 ± 1.4	141 ± 2.1	<0.001
Potassium, mmol/l	4.34 ± 0.40	4.32 ± 0.34	0.82
Chloride, mmol/l	105 ± 2.4	107 ± 2.2	<0.001
Anion gap, mmol/l	12 ± 2.1	11 ± 2.1	0.54
**Venous blood gas**			
pH	7.36 ± 0.03	7.34 ± 0.04	0.01
pCO_2_, kPa	6.41 ± 0.70	6.71 ± 0.78	0.04
pO_2_, kPa	3.3 (2.9–5.3)	3.3 (2.8–4.1)	0.28
HCO_3_^−^, mmol/l	26.9 ± 2.2	27.2 ± 2.3	0.45
Base excess, mmol/l	0.75 ± 1.9	0.76 ± 2.1	0.98
**Urinary parameters**			
24 hour urine volume, l	2.7 (1.7–3.3)	6.3 (4.4–7.2)	<0.001
Creatinine excretion, mmol/24u	12.0 (9.7–16.8)	12.2 (10.6–14.4)	0.69

Biochemical analyses are plasma concentrations. Data are presented as mean ± SD or median (interquartile range) as appropriate, and differences were tested using a paired *t*-test or Wilcoxon signed rank test, respectively. eGFR, estimated glomerular filtration rate, calculated with the Chronic Kidney Disease–Epidemiology Collaboration formula. Anion gap was calculated as: (plasma sodium + plasma potassium) – (plasma chloride + plasma HCO_3_^−^). Twenty-four hour urine volume and creatinine excretion was available in 21 patients, and plasma osmolality in 17 patients.

In this small, hypothesis-guided study, use of tolvaptan causes loss of circulatory volume, as illustrated by the increase in plasma osmolality, plasma sodium, and plasma chloride, which instigated a compensatory increase in copeptin. This indicates that there is an increased vasopressin secretion and thus potentially an increased V1 receptor activation. Evaluating these potential effects of V1 receptor activation, we did not observe a significant increase in plasma bicarbonate, our main outcome. Furthermore, we found no effect on HbA1c as marker for the glucose metabolism or on blood pressure. Several explanations for this unexpected finding can be offered. To start, the effect might be missed due to the short follow-up time of this study. In the TEMPO 3:4 trial, a significant effect on blood pressure developed gradually only after a prolonged time of treatment (J. E. Heida et al, manuscript under review, 2021). Furthermore, plasma bicarbonate was not measured with the gold-standard method, namely via arterial puncture, which could have limited the sensitivity of our investigation. Then again, prior research has shown that pH and bicarbonate measured in venous blood correlate adequately with their respective arterial values and can be used interchangeably.^6^ Therefore, an explanation for our observations is more likely to be found in changes in acid-base homeostasis induced by tolvaptan use than methodological issues.

We observe a shift toward a more acidotic state. We did not find differences in acid-base regulated metabolic factors, such as bicarbonate, base excess, and anion gap, that can account for this shift. Instead, we found an increase in pCO_2._ Prior studies provide a rationale for this observation: activation of the V1 receptor by vasopressin has also been linked to central respiratory rate control (Figure 1). Administration of vasopressin in dogs resulted in a higher pCO_2_ and decreased respiratory rate, whereas a V1 receptor antagonist had an inhibited effect.^7^^,^^8^ These findings were corroborated by experiments in another species, namely Sprague-Dawley rats.^9^ Therefore, in our patients with ADPKD, it could be that as result of V2 blockade, vasopressin increases, thereby activating more V1 receptors and thus resulting in a decreased respiratory rate and decreased pCO_2_. Although it certainly can be debated whether the venous pCO2 is a reliable reflection of its arterial value, the significant decrease in paired measurements of this arguably variable measure in this small cohort, together with the theoretical rationale, make this hypothesis more convincing.

**Figure 1:**
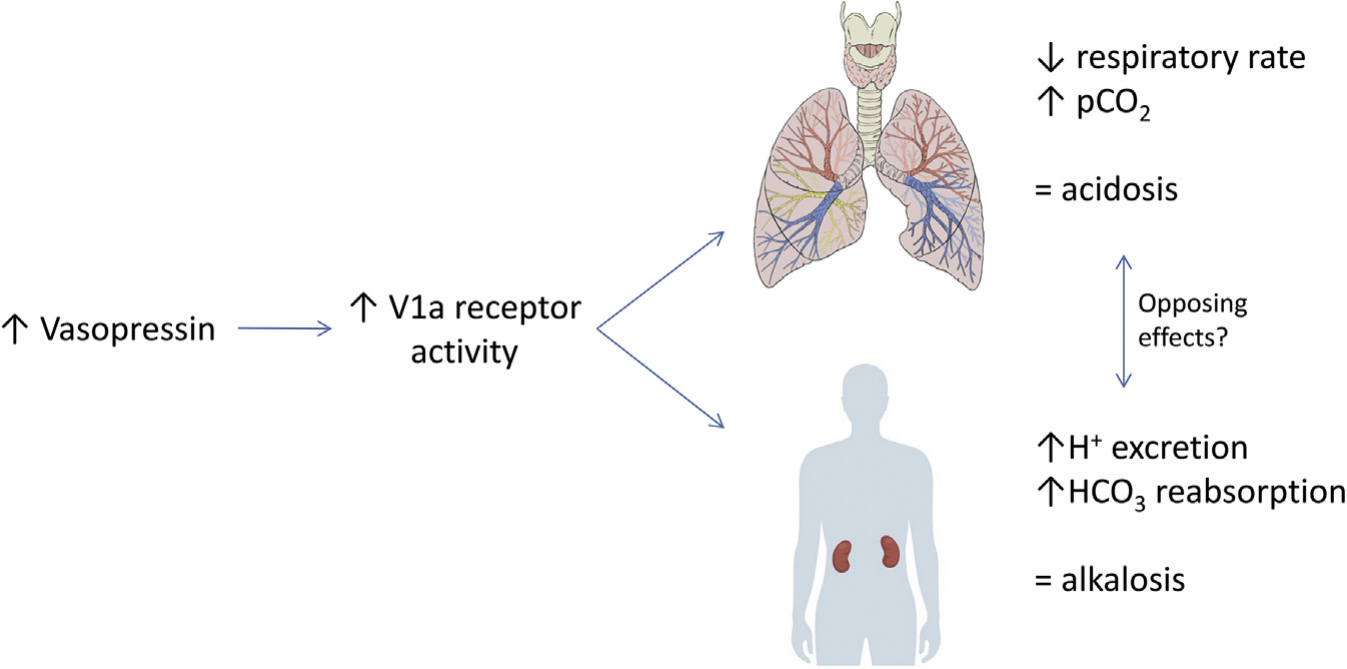
Effects of V1 receptor activation on acid-base homeostasis.

In summary, we hypothesized that prescription of a selective V2 receptor antagonist may induce a metabolic alkalosis as result of increasing levels of plasma vasopressin, thus enhancing V1 receptor–initiated reabsorption of bicarbonate by the kidneys. The collected data, however, contradict this hypothesis, given that no change in bicarbonate levels was found. In contrast, a slight but significant decrease in pH was observed under tolvaptan. This could be caused by an increased pCO2, but future research is necessary to further investigate this finding.

## Disclosures

RTG and EM have received consultancy fees from Otsuka Pharmaceutical Development & Commercialization (Rockville, MD), the manufacturer of tolvaptan. All money was paid to their institution. JEH discloses no conflicts of interests.
