# Supplementary material for: Acid-Base Homeostasis During Vasopressin V2 Receptor Antagonist Treatment in Autosomal Dominant Polycystic Kidney Disease Patients
Source: Kidney Int Rep. 2021 Jan 5;6(3):839–41. doi: 10.1016/j.ekir.2020.12.021 (PMC7938056; doi:10.1016/j.ekir.2020.12.021)
Supplement: Supplementary File (PDF) [file mmc1.pdf]

Supplementary methods

Study population

This is a prospective monocenter observational cohort study of Autosomal Dominant Polycystic Kidney Disease (ADPKD) patients who started with tolvaptan to slow disease progression. Clinical and laboratory data was collected between January 2018 and May 2020 during consecutive outpatient clinic visits to our ADPKD expertise center, the University Medical Center Groningen. Diagnosis of ADPKD was made by clinical evaluation of kidney cysts, as described by Ravine *et al*.[1] When treatment was indicated[2], tolvaptan was initiated at a split-dose regimen of 45 mg in the morning and 15 mg in the evening. After one month, tolerability and safety parameters were assessed and the dosage of tolvaptan was raised to 60/30 mg per day if possible for another month. The next month, the dosage was uptitrated to 90/30 mg per day if tolerated and a final visit was scheduled after one month to evaluate this highest dose.

Before start of data collection, we calculated that 31 patients would be needed to show a 1.0 mEq/L change in bicarbonate level assuming an alpha of 0.05, a beta of 0.20 and a standard deviation of 2.0.[3]

The protocol of this study was exempted of ethical approval by the institutional review board (M18.227802). Data has been collected with due consideration of the ICH-GCP (International Conference on Harmonization-Good Clinical Practice) guidelines.

Clinical data and Laboratory assessments

Clinical data included date of birth, biometric data and systolic and diastolic blood pressure blood pressure. Laboratory assessment included amongst others blood measurements of sodium, potassium, chloride, creatinine, HbA1c and venous pH, pO_2, pCO_2 , bicarbonate and base excess. These measurements were performed at the laboratory facility of our hospital (Roche Diagnostics, Indianapolis, United States). Venous blood gasses were measured separately by photometry at 37 degrees Celsius (ABL90 analyzer, Radiometer Benelux BV, The Netherlands). From these

measurements, anion gap was calculated as: (plasma sodium + plasma potassium) – (plasma chloride + plasma HCO_3^-). Creatinine was used to estimate the glomerular filtration rate with use of the CKD-EPI formula.[4] Copeptin was measured with use of a sandwich ELISA (ThermoFisher, Henningsdorf/Berlin, Germany).[5] In addition, the day before their visit patients collected 24 hour urine to assess the effect of tolvaptan on urinary output.

Imaging data

In case abdominal magnetic resonance images were available, total kidney volume was measured by manual tracing of T2-weighted coronal images by trained personnel (AnalyzeDirect, Inc., Overland Park, KS).[6] Thereafter, total kidney volume was adjusted for height and indexed for age to establish the Mayo htTKV risk class.[7]

Statistical analyses

Data are presented as mean ± standard deviation or median [interquartile range] as appropriate, and differences were tested using a paired t-test or Wilxocon signed rank test, respectively. Analyses were performed using SPSS (IBM Statistics version 22).

Image credits

Figure 1 is composed from images that were made available for reuse. Image of the lungs was made by Patrick J. Lynch, medical illustrator, and C. Carl Jaffe, MD, cardiologist.

References

1. Ravine D, Gibson RN, Walker RG, Sheffield LJ, Kincaid-Smith P, Danks DM. Evaluation of ultrasonographic diagnostic criteria for autosomal dominant polycystic kidney disease 1. *Lancet*. 1994;343(8901):824-827.

2. Gansevoort RT, Arici M, Benzing T, et al. Recommendations for the use of tolvaptan in autosomal dominant polycystic kidney disease: A position statement on behalf of the ERA-EDTA working groups on inherited kidney disorders and european renal best practice. *Nephrol Dial Transplant*. 2016;31(3):337-348.

3. Torres VE, Keith DS, Offord KP, Kon SP, Wilson DM. Renal ammonia in autosomal dominant polycystic kidney disease. *Kidney Int*. 1994;45(6):1745-1753.

4. Levey AS, Stevens LA, Schmid CH, et al. A new equation to estimate glomerular filtration rate. *Ann Intern Med*. 2009;150(9):604-612.

5. Morgenthaler NG, Struck J, Alonso C, Bergmann A. Assay for the measurement of copeptin, a stable peptide derived from the precursor of vasopressin. *Clin Chem*. 2006;52(1):112-119.

6. van Gastel MDA, Messchendorp AL, Kappert P, et al. T1 vs. T2 weighted magnetic resonance imaging to assess total kidney volume in patients with autosomal dominant polycystic kidney disease. *Abdom Radiol (NY)*. 2018;43(5):1215-1222.

7. Irazabal MV, Rangel LJ, Bergstralh EJ, et al. Imaging classification of autosomal dominant polycystic kidney disease: A simple model for selecting patients for clinical trials. *J Am Soc Nephrol*. 2015;26(1):160-172.
